# Transnasal Humidified Rapid Insufflation Ventilatory Exchange in Endoscopic Esophageal Surgery

**DOI:** 10.1177/00034894231216273

**Published:** 2023-12-05

**Authors:** Zao M. Yang, Tuan-Hsing Loh, Justin Ross, Kajal Dalal, Steffen E. Meiler, Gregory N. Postma

**Affiliations:** 1 Department of Otolaryngology, Head & Neck Surgery, UT Health San Antonio, San Antonio, TX, USA; 2 Department of Otolaryngology, Head & Neck Surgery, Augusta University Medical College of Georgia, Augusta, GA, USA; 3 Department of Anesthesiology, UT Health San Antonio, San Antonio, TX, USA; 4 Department of Anesthesiology, Augusta University Medical College of Georgia, Augusta, GA, USA

**Keywords:** THRIVE, high-flow nasal oxygen, apneic oxygenation, esophagus, airway management

## Abstract

**Objectives:**

Transnasal humidified rapid insufflation ventilatory exchange (THRIVE) describes apneic oxygenation using humidified high flow nasal-cannula oxygen. Although it has been described as a sole mode of oxygenation in endoscopic laryngotracheal surgery, its use in endoscopic esophageal surgery under general anesthesia with neuromuscular paralysis has not previously been described. The objective of this study is to assess the safety and efficacy of THRIVE in esophagology.

**Methods:**

We conducted a retrospective review of adult patients undergoing esophageal procedures under general anesthesia who were oxygenated using THRIVE at two academic institutions. Demographic, clinical, and anesthesiologic data were collected and analyzed.

**Results:**

14 cases performed from March 2021 to March 2022 met inclusion criteria. 13/14 (92.9%) of patients were able to maintain oxygenation throughout the entirety of their procedure. The mean apneic time was 17.9 minutes with a maximum of 32 minutes. One patient required “rescue” intubation due to failure to maintain oxygenation. Excluding the sole THRIVE failure, the median SpO_2_ at the conclusion of surgery was 99% (range 94-100%). A linear regression model yielded an increase in EtCO_2_ of 0.95 mmHg/min or 0.127 kPa/min. SpO_2_ was negatively associated with both tobacco pack-year smoking history (R^2^ = 0.343, *P* = .014) and BMI (R^2^ = 0.238, *P* = .038).

**Conclusion:**

THRIVE is a feasible, safe, and efficacious means of apneic oxygenation for patients undergoing esophageal endoscopic surgery under general anesthesia with neuromuscular paralysis, which may be particularly beneficial in patients with airway stenosis, as post-intubation changes can have severe clinical implications for this patient population. Obese patients and tobacco smokers may be at increased risk of oxygen desaturation when using THRIVE.

## Introduction

Transnasal humidified rapid insufflation ventilatory exchange (THRIVE) is a means of apneic oxygenation that can be utilized in airway management for airway and microlaryngeal surgery as a primary means of oxygenation in lieu of endotracheal intubation or jet ventilation.^1,2^ THRIVE has also been used for supplemental oxygenation in sedated esophageal procedures in the gastroenterology realm, though in these procedures patients are not paralyzed and are spontaneously breathing.^3^ For otolaryngologists, esophagoscopy typical utilizes rigid instrumentation and can involve concomitant laryngeal procedures, which relies on neuromuscular paralysis for exposure and procedural tolerance. The role of THRIVE for patients undergoing endoscopic esophageal surgery in this setting has not been studied. The objective of this study is to evaluate the efficacy and safety of THRIVE for airway management and oxygenation for patients undergoing esophageal endoscopic procedures under general anesthesia.

## Methods

A retrospective review was performed of patients who underwent endoscopic esophageal surgery with THRIVE for airway management at two institutions (UT Health San Antonio and the Medical College of Georgia at Augusta University) from March 2021 to March 2022. Institutional Review Board approval was obtained at both institutions. All patients were oxygenated using THRIVE under general anesthesia without an endotracheal tube in place. Demographic and clinical data were collected from the medical record including age, sex, body mass index (BMI), tobacco history, Charlson Comorbidity Index (CCI), American Society of Anesthesiologists (ASA) classification, surgical procedure performed, and surgical indication. Patients were excluded if there were insufficient anesthesiologic data or if the planned primary airway for the procedure involved endotracheal intubation.

Esophageal procedures in this cohort are performed in a hybrid rigid/flexible fashion. A Dedo laryngoscope is used to distend open the pharyngoesophageal segment (PES), which is otherwise collapsed when using flexible instrumentation alone, impairing examination and intervention to this area. The Dedo is then placed into suspension and a flexible esophagoscope is then used to perform the remainder of the evaluation of the esophagus and stomach, after which injection or dilation can be performed.

The airway protocol utilized for this study is outlined in Table 1. All procedures were performed in an operating room setting, and patients were preoxygenated while awake using THRIVE (Optiflow, Fisher & Paykel Healthcare) at 50 L/min. After induction of general anesthesia, patients were oxygenated using THRIVE at 70 L/min. Patients were then paralyzed with a nondepolarizing neuromuscular blocking agent and maintained on total intravenous anesthesia (TIVA) for the duration of the procedure. Intra-operative anesthetic records were used to obtain apneic times, oxygen saturation (SpO_2_), pre-apneic and post-apneic end-tidal carbon dioxide (EtCO_2_), and need for “rescue” intubation due to oxygen desaturation.

**Table 1.** THRIVE Airway Protocol.

1. Attach mask to patient and obtain seal. 2. Ask patient to take a deep breath. Record pre-apneic EtCO_2_. 3. Attach THRIVE nasal cannula to patient. Turn flow to 50 L/min. Preoxygenate for at least 3 minutes. 4. Induce patient. Turn THRIVE to 70 L/min. Start TIVA. 5. Temporarily remove nasal cannula to confirm ability to bag-mask after apnea in case of need for “rescue” ventilation. 6. Put nasal cannula back on. Mark start of apneic oxygenation time. 7. Paralyze patient and turn table to surgical team to perform procedure. 8. Record nadir SpO_2_ during case. If unable to maintain SpO_2_ > 90%, intubate with 5.0 ML tube. Record stop time and reason for THRIVE failure. 9. At conclusion of case, turn table back to anesthesia team, stop TIVA, and reverse paralysis. 10. Observe for return of spontaneous respiration. Mark end of apneic oxygenation time. 11. Obtain seal with mask to record post-apneic EtCO_2_.

Continuous variables are presented as mean (standard deviation) or median (range) for non-parametric data. The Pearson correlation coefficient was calculated to determine correlation between demographic and clinical data and tolerance of THRIVE as measured by apneic time, lowest SpO_2_, and post-apneic EtCO_2_. Statistical significance was determined using a level of α = .05.

## Results

In all, 14 patients underwent esophageal endoscopic surgery under general anesthesia with THRIVE for oxygenation (Table 2). Eight (57%) were men and six (43%) were women. The average age was 66.8 (13.0) and average BMI was 24.3 (5.4). In all, 12 patients (85.7%) had concomitant balloon dilation of the PES. Indications for dilation included post-radiation esophageal stenosis, primary cricopharyngeus muscle dysfunction, a PES web, and inclusion body myositis. Two patients (14.3%) had concomitant laryngeal intervention with suspension micro-laryngoscopy. One patient underwent esophagoscopy as a component of panendoscopy for workup and diagnosis of a new laryngeal primary head and neck cancer, and one underwent esophagoscopy for surveillance examination of a previously endoscopically resected liposarcoma of the hypopharynx/proximal esophagus.

**Table 2.** Patient Characteristics and Clinical Data.

Age	BMI	Sex	Tobacco history (pack-years)	CCI	ASA	Procedure	Indication	Post-apneic EtCO_2_	Apneic time	Nadir SpO_2_
31	23.6	F	0	0	2	Esophagoscopy with balloon dilation	PES web	39	21	100
54	21.0	M	5	4	3	Esophagoscopy with balloon dilation	Cricopharyngeus muscle dysfunction	41	21	100
60	24.6	M	0	3	3	Esophagoscopy with balloon dilation	Post-radiation esophageal stenosis	34	7	98
62	34.7	F	66	2	3	Esophagoscopy	Hypopharyngeal liposarcoma, surveillance exam	35	10	100
66	29.7	M	0	6	3	Esophagoscopy with balloon dilation, micro-laryngoscopy	Inclusion body myositis, vocal fold atrophy	62	32	99
69	26.6	M	50	5	3	Esophagoscopy, micro-laryngoscopy	Laryngeal cancer	50	31	94
69	20.6	F	0	2	3	Esophagoscopy with balloon dilation	Post-radiation esophageal stenosis	70	32	97
69	23.2	M	0	2	3	Esophagoscopy with balloon dilation	Post-radiation esophageal stenosis	56	14	98
70	35.0	M	90	4	3	Esophagoscopy with balloon dilation	PES web	53	5	88
71	20.0	M	60	3	3	Esophagoscopy with balloon dilation	Post-radiation esophageal stenosis, posterior glottic stenosis	53	26	99
72	20.8	M	30	4	3	Esophagoscopy with balloon dilation	Post-radiation esophageal stenosis	45	11	99
77	22.8	F	0	5	3	Esophagoscopy with balloon dilation	Post-radiation esophageal stenosis	35	8	99
81	19.4	F	0	6	3	Esophagoscopy with balloon dilation	Post-radiation esophageal stenosis	31	11	97
84	18.5	F	0	5	3	Esophagoscopy with balloon dilation	Cricopharyngeus muscle dysfunction	34	22	100

In our cohort 13/14 (92.9%) of patients were able to maintain oxygenation throughout the entirety of their procedure. The mean apneic time was 17.9 minutes with a maximum of 32 minutes. One patient required “rescue” intubation due to failure to maintain SpO_2_ after 5 minutes of THRIVE. There was a corresponding EtCO_2_ increase of 18. This patient was intubated without difficulty at a nadir SpO_2_ of 88%, and the remainder of the procedure was completed without complication. This patient had a BMI of 35 and a smoking history of 90 pack-years, both of which were maximum values in our study cohort. Excluding the sole THRIVE failure, the median SpO_2_ at the conclusion of surgery was 99% (range 94-100%).

**Figure 1.**
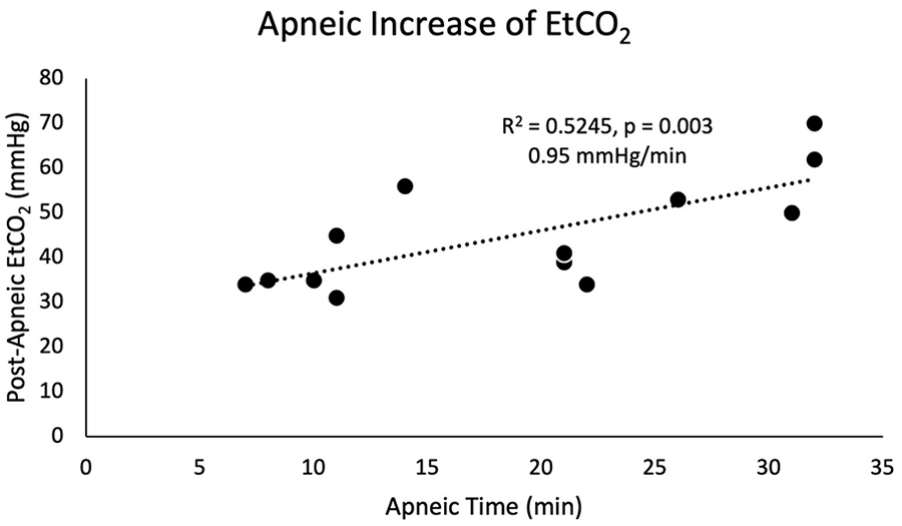
Duration of apnea was associated with a statistically significant increase in EtCO_2_. A linear regression model demonstrates an increase in EtCO_2_ of 0.95 mmHg/min.

Three patients were missing data for pre-apneic capnography. For the remainder of cohort, mean pre-apneic EtCO_2_ was 30.5 (5.2). Mean post-apneic EtCO_2_ for the entire cohort was 45 (12.3). The average change in EtCO_2_ for the patients with available pre-apneic capnography data was 15.2 (10.9). 36.4% had an EtCO_2_ increase of <10. There was a statistically significant increase in post-apneic EtCO_2_ with increased apneic time (R^2^ = 0.524, *P* = .003; Figure 1). After excluding the THRIVE failure, a linear regression model yielded an increase in EtCO_2_ of 0.95 mmHg/min or 0.127 kPa/min.

**Figure 2.**
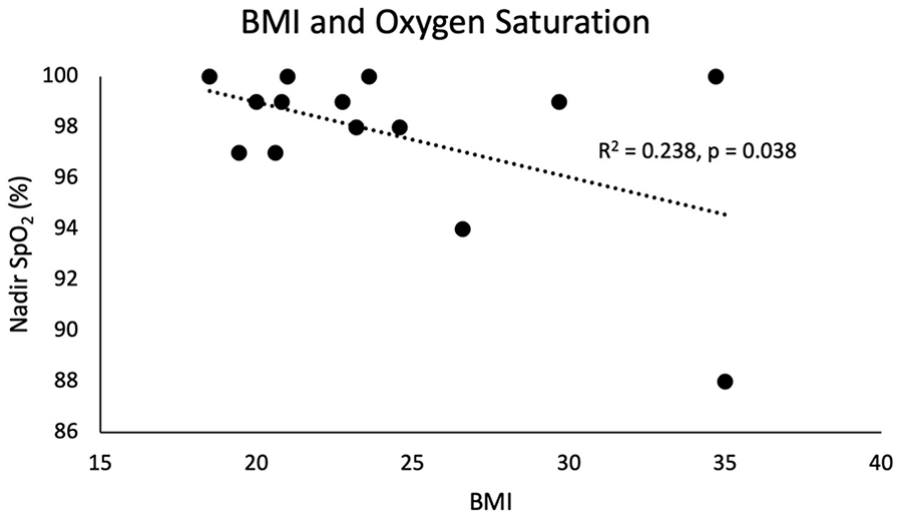
Patients with a higher BMI were associated with a statistically significant lower nadir SpO_2_.

**Figure 3.**
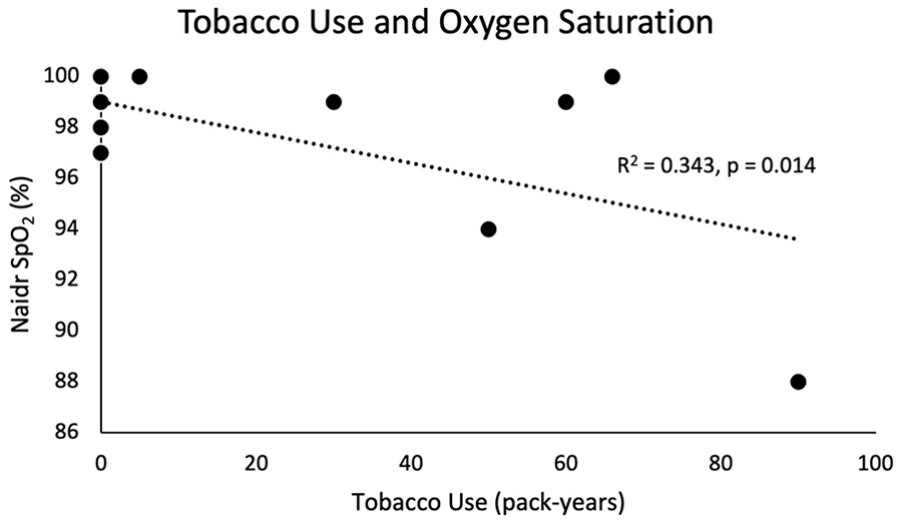
Tobacco use in pack-years was associated with a statistically significant lower nadir SpO_2_.

One patient had an ASA classification of 2 (7.1%) and the remainder (92.9%) had an ASA classification of 3. CCI ranged from 0 to 6 with a median of 4. There was no association between age, CCI, or ASA classification with lowest SpO_2_ or post-apneic EtCO_2_. Patient nadir SpO_2_ was negatively associated with both BMI (R^2^ = 0.238, *P* = .038; Figure 2) and tobacco pack-year smoking history (R^2^ = 0.343, *P* = .014; Figure 3). However, there was no association with either tobacco history or BMI with post-apneic EtCO_2_.

## Discussion

THRIVE has been demonstrated as a safe means for airway management during microlaryngeal and endoscopic airway surgery^2,4-6^ and its use has become increasingly popular in laryngology as it obviates the necessity for endotracheal intubation during these cases. In endoscopic laryngotracheal surgery, the PES is typically collapsed due to resting closure of the cricopharyngeus muscle, which limits flow of oxygen into the esophagus with preferential shunting into the airway. Although distension of the PES with a rigid laryngoscope during hybrid esophageal intervention may potentially alter airflow dynamics during THRIVE, data from this retrospective study indicate that THRIVE is a feasible, safe, and efficacious means of apneic oxygenation for patients undergoing esophageal endoscopic surgery under general anesthesia with neuromuscular paralysis. The overwhelming majority of patients in our cohort were able to successfully maintain oxygen saturation throughout the entirety of their surgical procedure, with apnea times over 30 minutes.

As expected, duration of apnea was associated with a statistically significant increase in EtCO_2_ at case conclusion. Linear regression models from our cohort suggest that EtCO_2_ increases at a rate of approximately 0.95 mmHg/min or 0.127 kPa/min of apnea while under THRIVE for esophageal endoscopic intervention (Figure 1), which is comparable to other similar studies for laryngotracheal intervention, which range from 0.11 to 0.17 kPa/min.^1,2,4,6^ The combined weighted average of these studies with our cohort yields a rate of change of 0.13 kPa/min, or approximately 1 mmHg/min of apnea when using THRIVE for upper aerodigestive tract cases. THRIVE lowers the rate of development of hypercapnia, which ranges from 0.35 to 0.45 kPa/min in apneic patients without THRIVE.^7^ This improved carbon dioxide clearance may be driven by turbulent gaseous vortices in the airway that are generated from the high flow of oxygen.^8^

The increase in EtCO_2_ in our cohort was not associated with a lower nadir SpO_2_, and patients were able to maintain oxygenation on THRIVE despite worsening hypercapnia. Clinical factors such as BMI and tobacco history did not appear to affect the rate of increase of EtCO_2_ during apnea but were associated with oxygen desaturation. Obese patients were more likely to have a lower nadir SpO_2_ when undergoing apneic esophageal endoscopy with THRIVE (Figure 2). This is congruent with existing literature that suggests BMI is a potential prognostic indicator for THRIVE failure in patients undergoing endoscopic surgical intervention in the larynx and proximal trachea.^9^ A similar effect was noted in patients with a more substantial history of tobacco use (Figure 3). Our data suggest that obese patients and smokers are more likely to experience failure to maintain oxygenation when using THRIVE.

THRIVE may be a preferred strategy for airway management during quick esophageal cases that are expected to last approximately 30 minutes or less. Use of THRIVE avoids airway trauma related to intubation, which can be particularly beneficial in patients who have concomitant airway disease such as posterior glottic or subglottic stenosis. Post-intubation changes can have severe clinical implications for this patient population, and some patients with airway stenosis may not tolerate intubation. The authors recommend consideration of THRIVE as an airway management technique when endoscopic esophageal intervention is planned for patients with posterior glottic or subglottic stenosis.

THRIVE can also be used when endoscopic intervention is planned for both the airway and the esophagus. When used in endoscopic laryngotracheal surgery, THRIVE has been shown to improve operative time and anesthesia time^10^ and even improved post-operative pain.^11^ This was not directly examined in our study, though the average total apnea time was only 18 minutes in our cohort. The authors additionally noted that THRIVE allows for improved access and visualization owing to the lack of an endotracheal tube. THRIVE is not ideal for cases that are expected to last longer than 30 minutes or in patients who are expected to have poor pulmonary reserve such as smokers or obese patients.

As a proof-of-concept study, our cohort consisted of a relatively small sample size. Other limitations include those inherent to retrospective studies, such as a lack of a randomized comparison with intubated patients. Additional study is warranted in patients undergoing endoscopic esophageal procedures under general anesthesia with neuromuscular paralysis.

## Conclusion

THRIVE appears to be a feasible, safe, and efficacious means of apneic oxygenation for patients undergoing esophageal endoscopic surgery under general anesthesia with neuromuscular paralysis. THRIVE may be of particular benefit in patients who would benefit from avoidance of endotracheal intubation, such as patients with concomitant laryngotracheal stenosis. Obese patients and tobacco smokers may be at increased risk of oxygen desaturation when using THRIVE.
